# Origin of an Anticrossing between a Leaky Photonic Mode and an Epsilon-Near-Zero Point of Silver

**DOI:** 10.1021/acs.jpcc.2c05836

**Published:** 2022-11-03

**Authors:** Wai Jue Tan, Philip A. Thomas, William L. Barnes

**Affiliations:** Department of Physics and Astronomy, University of Exeter, ExeterEX4 4QL, U.K.

## Abstract

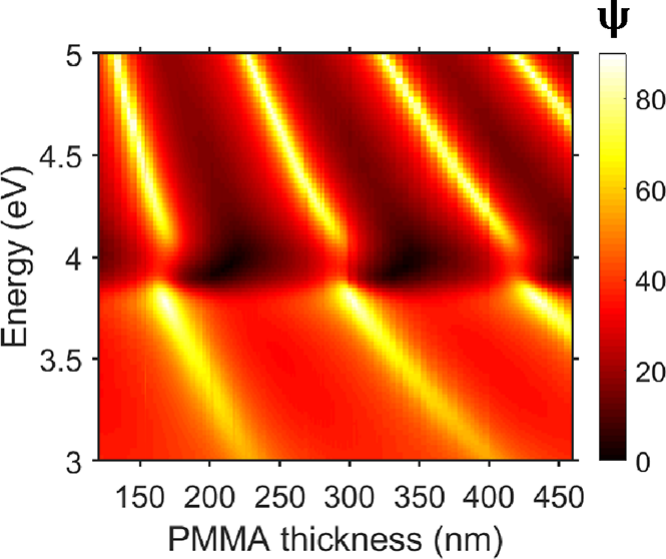

Strong light–matter coupling hybridizes light and matter to form states known as polaritons, which give rise to a characteristic anticrossing signature in dispersion plots. Here, we identify conditions under which an anticrossing can occur in the absence of strong coupling. We study planar silver/dielectric structures and find that, around the epsilon-near-zero point in silver, the impedance matching between the silver and dielectric layers gives rise to an anticrossing. Our work shows that care must be taken to ensure that anticrossing arising from impedance matching is not misattributed to strong coupling.

## Introduction

Epislon-near-zero (ENZ) materials are a class of materials that have dielectric permittivities ε = ε′ + *i*ε^″^ with real components ε′ that cross zero at one or more energies. They have an array of unusual properties: tunneling through narrow, distorted channels; reshaping the radiation pattern of a source; and the enhancement of nonlinear optical interactions.^1−5^ In highly subwavelength structures, at energies where ε′ → 0, they can support localized surface modes called ENZ modes. ENZ modes are coupled surface modes that can only be optically excited by TM polarized light that has an in-plane momentum greater than light in free space. Therefore, coupling with ENZ modes often requires nanostructures that scatter light to gain sufficient momentum to match that of the ENZ mode. ENZ materials can generate large electric-field enhancements, making these materials promising candidates for applications such as sensing, light concentration, and enhancement of molecular emissions.^6−8^

ENZ materials have also found use in strong light–matter coupling experiments. Strong coupling occurs when the rate of interaction between two resonators is greater than the rate of dissipation.^9−11^ In this regime, the two resonators will hybridize to form two new coupled states that inherit the properties of both uncoupled resonators.^12^ Strong coupling has been studied in a variety of systems, such as atoms in a cavity, quantum dots in a photonic crystal, and molecules close to plasmonic structures.^1,13,14^ Some ENZ materials possess electrically tuneable ENZ modes in the near-infrared.^15^ ENZ materials, therefore, allow for the electrical modulation of strong coupling and enhanced light–matter interactions at wavelengths important for telecommunication applications.^15−18^

One challenge in light–matter coupling experiments is the assured determination of whether or not a system lies in the strong coupling regime.^19^ A characteristic feature of strong coupling is an anticrossing between the dispersions of the two resonators; the energy splitting between the two anticrossed modes (known as the Rabi splitting) is often used to quantify the extent of strong coupling.^10^ However, the splitting of resonances and the appearance of an anticrossing can be caused by other phenomena, such as surface-enhanced absorption^20^ or an inappropriate choice of focusing optics.^21^ The observed Rabi splitting also varies depending on the choice of measurement technique,^22^ and misleading optical signatures can appear in transient absorption spectroscopy.^23^ In this paper, we show that anticrossing can be observed around the ENZ point in silver. We show that this anticrossing feature arises from the impedance matching of silver around its ENZ point to an adjacent dielectric film and is not due to strong coupling. Our work shows that, when studying strong coupling, care must be taken to ensure that anticrossing arising from impedance matching is not misattributed to strong coupling.

## Methods

### Sample Fabrication

Ag films (thickness: 25 nm) and Al films (thickness: 10 nm) were deposited on glass substrates using thermal evaporation. Both metals were deposited at a rate of 0.1–0.2 nm s^–1^. Poly(methyl methacrylate) (PMMA) films were deposited on the metal films by spin-coating a 4 wt % solution of PMMA in anisole (PMMA 950k A4). To obtain films with a wide range of thicknesses (100–500 nm), a small volume (∼10 μL) of PMMA solution was drop-cast slightly off-center of the substrate and spun at a wide range of spin speeds, from 1500 rpm (revolutions per minute) to 9000 rpm (see Supporting Information Figure S1). The thicknesses of the PMMA layers were determined through ellipsometric fitting.

### Ellipsometry

Ellipsometry measures the complex reflection coefficient ratio of p- and s-polarized light, expressed as ρ in terms of the amplitude Ψ and phase Δ: 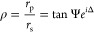
1where *r*_p_ and *r*_s_ are the Fresnel reflection coefficient of p- and s-polarized light, respectively, tan Ψ is the amplitude ratio |ρ|, and Δ is the phase difference between p- and s-polarized light. Ellipsometry is an ideal measurement tool for our experiment since it allows us to concurrently study both TE modes (maxima in Ψ) and TM modes (minima in Ψ) in our structures. Our ellipsometer (J. A. Woollam Co. M-2000X) has a spectral range of 0.7 eV < *E* < 5.8 eV, and since ellipsometry measures the ratio of two quantities it is a low-noise measurement technique that allows us to clearly characterize the UV response of our structure. We used focusing optics to measure small regions (∼100 × 200 μm^2^) of our samples over which the PMMA film was approximately smooth (see Supporting Information Figure S1b).

## Results and Discussion

**Figure 1:**
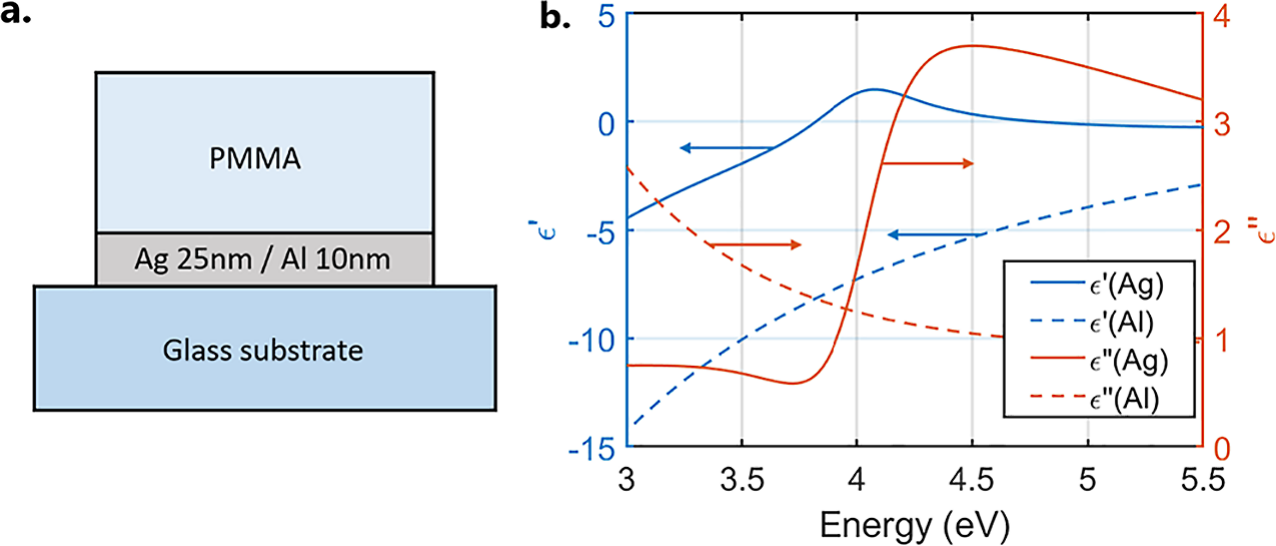
(a) Schematic of the system studied: PMMA on top of the Ag/Al film of thickness 25 nm/10 nm on a glass substrate. (b) Complex permittivities of Ag and Al, obtained through fitting to ellipsometry data.

We studied thin metal films covered by a dielectric layer (schematic in Figure 1a). The optical constants of Ag and Al are shown in Figure 1b, and the optical constants of PMMA are shown in Supporting Information Figure S2. These optical constants were determined using spectroscopic ellipsometry. A Drude–Lorentz model was used for the Ag and Al films, while a Cauchy dielectric model was fit to the PMMA data. The highly reflective metal mirror, together with the huge contrast in the refractive index between the PMMA layer and air, allows this structure to support leaky modes.^24^ Leaky modes are confined within the dielectric layer, but unlike Fabry–Perot modes (which have a low-field amplitude at the dielectric layer’s interfaces) the leaky mode field amplitude is minimal at the metal/dielectric interface and maximum at the air/dielectric interface.^25^ This confined field is sufficient for strong and ultrastrong coupling.^24,25^ Ag has two ENZ points in the ultraviolet spectrum at energies of *E* = 3.8 eV and *E* = 4.9 eV caused by interband transitions. We repeated our experiments, replacing Ag with Al, which has no ENZ points in this spectral region, as a control sample.

**Figure 2:**
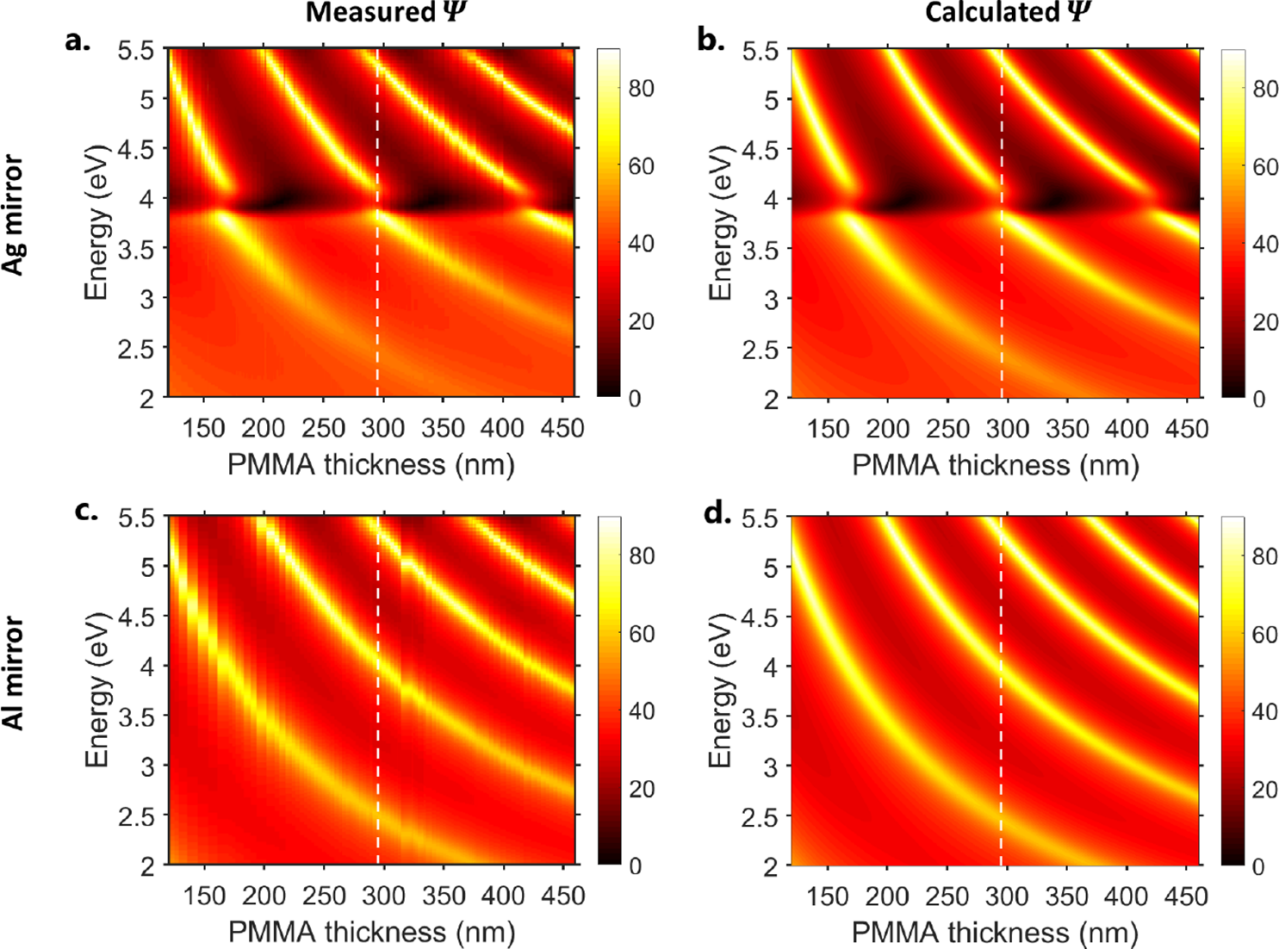
Measured (a,c) and calculated (b,d) ellipsometry amplitude parameter Ψ of the structure shown in Figure 1a. Panels (a,b) are for silver, while panels (c,d) are for aluminum.

**Figure 3:**
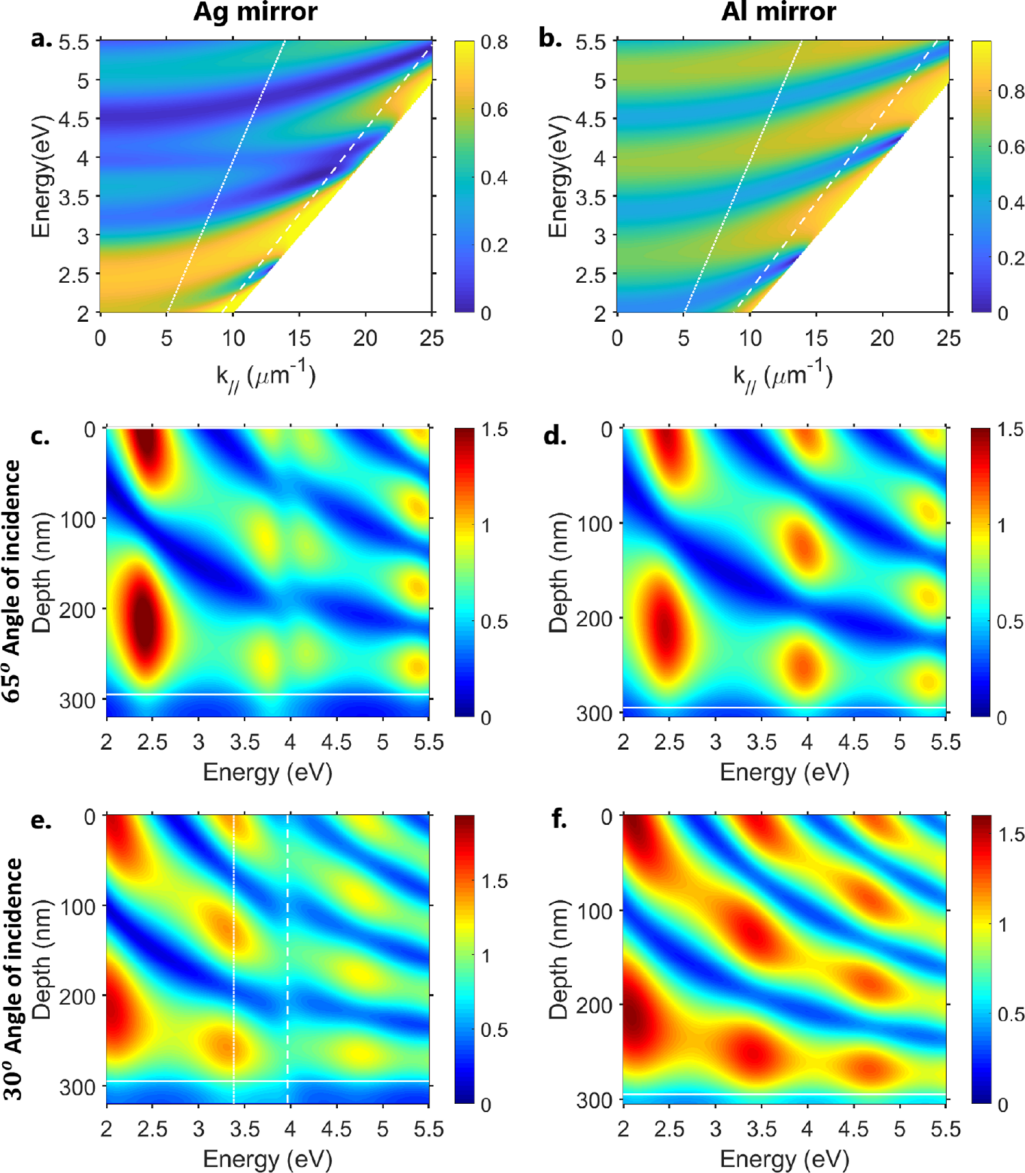
Calculated TE reflection spectrum of (a) glass/Ag (25 nm)/PMMA (295 nm) and (b) glass/Al (10 nm)/PMMA (295 nm) as a function of energy and in-plane wave-vector *k*_∥_ = 2π/λ sin θ, where λ is the wavelength of light. Slanted white dotted lines indicate θ = 30°, while the slanted white dashed lines indicate θ = 65°. (c–f) Field profile of the (c) Ag mirror structure at θ *=* 65°, (d) Al mirror structure at θ = 65°, (e) Ag mirror structure at θ = 30°, and (f) Al mirror structure at θ = 30°. Horizontal white solid lines in the field profile indicate the metal/PMMA interface. Vertical white lines indicate the energy of the reflection minima at θ = 30° around the ENZ point of Ag.

We characterized our samples using ellipsometry. Simultaneously, studying both TE and TM leaky modes provides another control since we only expect ENZ modes to interact with TM light modes.^26^Figure 2 shows the Ψ spectra of PMMA films covering the (a) Ag mirror and (c) Al mirror for PMMA thicknesses ranging from 120 to 460 nm, measured at an incident angle of θ = 65°. These results match well with Fresnel transfer matrix calculations (Figure 2b,d). In all structures, there is a clear set of TE and TM leaky modes. The TE leaky modes (maxima in Ψ) are clearer than the lossier TM modes (minima in Ψ). The TE modes in the Ag mirror structure display a clear anticrossing around 3.98 eV. In Figure 3a,b, we plot the calculated TE reflection dispersion profiles for structures with a PMMA film thickness of 295 nm (corresponding to the dashed lines in the plots in Figure 2). The TM reflection dispersion profiles are plotted in Supporting Information Figure S3, and both the TE and TM absorption are plotted in Supporting Information Figure S4. We also observe anticrossing at around 3.98 eV for the Ag/PMMA structure but not for the Al/PMMA structure, confirming that the anticrossing in Figure 2 is not just an artifact of ellipsometry. A clearer anticrossing can be seen in Supporting Information Figure S5, where the TE reflection dispersion profile is calculated for the similar structure but with PMMA film thickness of 270 nm. In the Ag/PMMA dispersion plot, a reflection minimum persists at 3.98 eV at lower incident angles (i.e., lower wavevector), far away from the point of anticrossing. No equivalent feature exists in the Al/PMMA dispersion plot. Al is a lossier metal than Ag, meaning that the leaky modes in the Ag/PMMA structure are of a higher quality than the modes supported by the Al/PMMA structure. This is clear in both Figures 2 and 3. The difference is most striking for lower wavevectors in Figure 3a,b; at higher incident angles, the TE field strengths are comparable. In Figure 3c,d, we compare the TE field profiles for Ag/PMMA and Al/PMMA structures at θ = 65°: the only significant difference is that the field enhancement peaks that occur at around 3.98 eV are split in the Ag/PMMA case, while they remain unsplit for Al/PMMA. There is no mode splitting at θ = 30° (Figure 3e,f), where the peaks in the electric field have been detuned from 3.98 eV. We do, however, observe a slight perturbation of the field profile around 3.98 eV in the Ag/PMMA structure that is not present in the Al/PMMA structure.

It is tempting to attribute this anticrossing behavior to the ENZ properties of Ag, since this anticrossing (at 3.98 eV) occurs at a similar energy to an ENZ point in Ag (at 3.8 eV). However, we observed anticrossing with TE-polarized light, and ENZ materials usually only interact with TM-polarized light. In most studies of strong coupling with ENZ materials, ENZ modes are involved.^15,27,28^ ENZ modes are surface modes that occur in deep-subwavelength films; such surface modes can only couple to *p*-polarized light with momentum greater than that of free-space light.^26,29^ Within the radiative region, thin ENZ films can also support Berreman modes.^29−31^ Berreman modes provide strong out-of-plane electric-field enhancement due to the continuity of the normal component of ; therefore, the excitation of Berreman modes also requires TM-polarized light. Longitudinal resonances can exist around the ENZ point of a material with low loss.^32^ The longitudinal resonance arises due to the presence of surface charge, which also requires electric field normal to the surface to excite it. Furthermore, Ag possesses a second ENZ point at 4.6 eV, at which no anticrossing or absorption is observed. Therefore, since our system is a simple multilayer planar structure, and anticrossing is observed in the TE leaky mode, we can rule out ENZ modes, Berreman modes, and longitudinal resonance as origins of this phenomenon.

We can also rule out molecular modes in PMMA as the source of anticrossing. While PMMA possesses vibrational modes that allow it to couple to midinfrared light,^33^ it possesses negligible absorption features around 4 eV.

**Figure 4:**
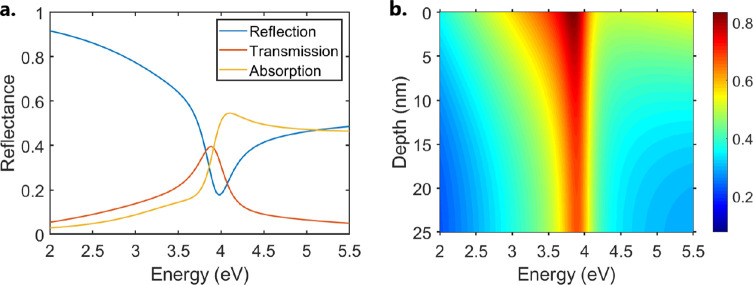
(a) Calculated TE reflection (blue), transmission (red), and absorption (yellow) spectra of the PMMA superstrate/Ag (25 nm)/glass substrate structure at 65^°^. (b) TE electric field profile of the PMMA superstrate/Ag (25 nm)/glass substrate at 65^°^.

Instead, we suggest a simpler explanation for the observed anticrossing. Between silver’s two ENZ points at 3.8 and 4.9 eV, the real part of its permittivity is positive, providing a spectral region where silver stops behaving as a metal. This small window of nonmetallic behavior allows light to propagate within the silver. The impedance mismatch between silver and PMMA reaches a minimum at 3.98 eV, allowing most light in the PMMA layer to transmit into the silver layer. This can be seen in Figure 4. Panel a shows the calculated reflection spectrum of a 25 nm silver film on a glass substrate, with the incident light entering from PMMA. The reflection minimum at 3.98 eV corresponds to the point of minimal impedance mismatch between PMMA and silver. Panel b shows the TE electric field profile inside the silver layer, clearly showing that the reflection minimum in panel a corresponds to the point at which the electric field cannot be confined to form a leaky mode, resulting in a splitting at around 3.98 eV.

**Figure 5:**
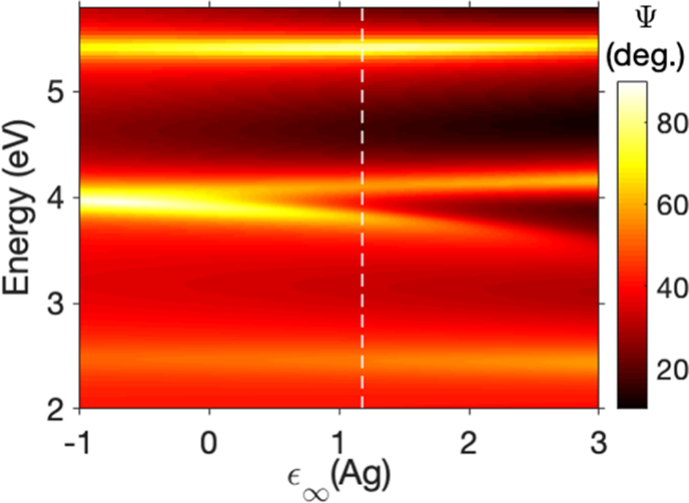
Effect of modifying the background permittivity ε_∞_ of Ag on Ψ, calculated for the Ag(25 nm)/PMMA(291 nm) structure at θ = 65°. Vertical dashed line shows the experimentally derived value of ε_∞_ for Ag.

To confirm that the impedance matching between Ag and PMMA is the source of anticrossing, we recalculated the Ψ spectra while varying ε_∞_, the background permittivity for Ag. Background permittivity is a constant independent of frequency: modifying it changes the real permittivity of Ag for all energies by the same quantity, which in turn modifies the impedance mismatch between the Ag and PMMA. We plot the results from these calculations (for −1 ≤ ε_∞_ ≤ 3) in Figure 5. The experimentally derived value of ε_∞_ = 1.18 for Ag is indicated by the vertical dashed line. For the lowest values of ε_∞_ < 0, the impedance mismatch between Ag and PMMA is high enough to allow a leaky mode to be supported at around 4 eV, similar to the higher-order leaky mode at 5.4 eV and the lower-order mode at 2.5 eV. As ε_∞_ turns positive, the impedance mismatch at around 4 eV becomes low, and the leaky mode can no longer be supported, resulting in splitting. This behavior strongly resembles the transition from the weak to a strong coupling regime as the coupling strength between light and matter states is increased,^19^ yet here it is simply a result of improved impedance matching. A similar calculation with the TE reflectivity spectra can be found in Supporting Information Figure S6, and the reflectivity dispersion for different values of ε_∞_ together with the Ag refractive indices can be found in Supporting Information Figure S7.

## Conclusions

In conclusion, we have shown that the leaky modes supported in an Ag/PMMA structure show an anticrossing around the ENZ point of the silver film. Anticrossing occurs in TE leaky modes which show that it is not a result of the ENZ properties of the silver film. We have shown that the anticrossing is not a result of strong coupling between the leaky mode and any resonant state but the impedance matching between the Ag and PMMA. Our results highlight the unusual optical signatures that can arise around ENZ points and that the observation of anticrossing is not by itself a guarantee of strong light–matter coupling.

## Supporting Information Available

Sample design; optical constants of PMMA; calculated TM reflection and TE and TM absorption spectra of glass/metal/PMMA (295 nm) structures as a function of in-plane wave-vector; calculated TE reflection spectrum of the glass/Ag (25 nm)/PMMA (270 nm) structure as a function of in-plane wave-vector; and the effect of modifying the background permittivity ε_∞_ of Ag on the TE reflectivity of the Ag(semiinfinite)/PMMA (291 nm) structure and PMMA/Ag interface (PDF)

## Notes

Research data supporting this publication are openly available from the University of Exeter’s institutional repository: https://doi.org/10.24378/exe.4284.

## References

[ref1] Chikkaraddy R.; de Nijs B.; Benz F.; Barrow S. J.; Scherman O. A.; Rosta E.; Demetriadou A.; Fox P.; Hess O.; Baumberg J. J. Single-molecule strong coupling at room temperature in plasmonic nanocavities. Nature 2016, 535, 127–130. 10.1038/nature17974. 27296227 PMC4947385

[ref2] Silveirinha M.; Engheta N. Tunneling of Electromagnetic Energy through Subwavelength Channels and Bends using εε-Near-Zero Materials. Phys. Rev. Lett. 2006, 97, 157403 10.1103/physrevlett.97.157403. 17155357

[ref3] Edwards B.; Alù A.; Young M. E.; Silveirinha M.; Engheta N. Experimental Verification of Epsilon-Near-Zero Metamaterial Coupling and Energy Squeezing Using a Microwave Waveguide. Phys. Rev. Lett. 2008, 100, 033903 10.1103/PhysRevLett.100.033903. 18232982

[ref4] Alù A.; Silveirinha M. G.; Salandrino A.; Engheta N. Epsilon-near-zero metamaterials and electromagnetic sources: Tailoring the radiation phase pattern. Phys. Rev. B: Condens. Matter Mater. Phys. 2007, 75, 155410 10.1103/physrevb.75.155410.

[ref5] Argyropoulos C.; Chen P.-Y.; D’Aguanno G.; Engheta N.; Alù A. Boosting optical nonlinearities in ε-near-zero plasmonic channels. Phys. Rev. B: Condens. Matter Mater. Phys. 2012, 85, 045129 10.1103/physrevb.85.045129.

[ref6] Alù A.; Engheta N. Light squeezing through arbitrarily shaped plasmonic channels and sharp bends. Phys. Rev. B: Condens. Matter Mater. Phys. 2008, 78, 035440 10.1103/physrevb.78.035440.

[ref7] Alù A.; Engheta N. Dielectric sensing in ε-near-zero narrow waveguide channels. Phys. Rev. B: Condens. Matter Mater. Phys. 2008, 78, 045102 10.1103/physrevb.78.045102.

[ref8] Alù A.; Engheta N. Boosting Molecular Fluorescence with a Plasmonic Nanolauncher. Phys. Rev. Lett. 2009, 103, 043902 10.1103/PhysRevLett.103.043902. 19659353

[ref9] Lidzey D. G.; Bradley D. D. C.; Skolnick M. S.; Virgili T.; Walker S.; Whittaker D. M. Strong exciton–photon coupling in an organic semiconductor microcavity. Nature 1998, 395, 53–55. 10.1038/25692.

[ref10] Törmä P.; Barnes W. L. Strong coupling between surface plasmon polaritons and emitters: a review. Rep. Prog. Phys. 2015, 78, 013901 10.1088/0034-4885/78/1/013901. 25536670

[ref11] Ebbesen T. W. Hybrid light–matter states in a molecular and material science perspective. Acc. Chem. Res. 2016, 49, 2403–2412. 10.1021/acs.accounts.6b00295. 27779846

[ref12] Lidzey D. G.; Bradley D. D. C.; Virgili T.; Armitage A.; Skolnick M. S.; Walker S. Room temperature polariton emission from strongly coupled organic semiconductor microcavities. Phys. Rev. Lett. 1999, 82, 3316–3319. 10.1103/physrevlett.82.3316.

[ref13] McKeever J.; Boca A.; Boozer A. D.; Buck J. R.; Kimble H. J. Experimental realization of a one-atom laser in the regime of strong coupling. Nature 2003, 425, 268–271. 10.1038/nature01974. 13679909

[ref14] Yoshie T.; Scherer A.; Hendrickson J.; Khitrova G.; Gibbs H. M.; Rupper G.; Ell C.; Shchekin O. B.; Deppe D. G. Vacuum Rabi splitting with a single quantum dot in a photonic crystal nanocavity. Nature 2004, 432, 200–203. 10.1038/nature03119. 15538363

[ref15] Jun Y. C.; Reno J.; Ribaudo T.; Shaner E.; Greffet J.-J.; Vassant S.; Marquier F.; Sinclair M.; Brener I. Epsilon-near-zero strong coupling in metamaterial-semiconductor hybrid structures. Nano Lett. 2013, 13, 5391–5396. 10.1021/nl402939t. 24124754

[ref16] Campione S.; Wendt J. R.; Keeler G. A.; Luk T. S. Near-infrared strong coupling between metamaterials and epsilon-near-zero modes in degenerately doped semiconductor nanolayers. ACS Photonics 2016, 3, 293–297. 10.1021/acsphotonics.5b00663.

[ref17] Hendrickson J. R.; Vangala S.; Dass C.; Gibson R.; Goldsmith J.; Leedy K.; Walker D. E. Jr; Cleary J. W.; Kim W.; Guo J. Coupling of epsilon-near-zero mode to gap plasmon mode for Flat-Top wideband perfect light absorption. ACS Photonics 2018, 5, 776–781. 10.1021/acsphotonics.7b01491.

[ref18] Passler N. C.; Gubbin C. R.; Folland T. G.; Razdolski I.; Katzer D. S.; Storm D. F.; Wolf M.; De Liberato S.; Caldwell J. D.; Paarmann A. Strong coupling of epsilon-near-zero phonon polaritons in polar dielectric heterostructures. Nano Lett. 2018, 18, 4285–4292. 10.1021/acs.nanolett.8b01273. 29894195

[ref19] Thomas P. A.; Tan W. J.; Fernandez H. A.; Barnes W. L. A new signature for strong light-matter coupling using spectroscopic ellipsometry. Nano Lett. 2020, 20, 6412–6419. 10.1021/acs.nanolett.0c01963. 32709208 PMC7608940

[ref20] Zengin G.; Gschneidtner T.; Verre R.; Shao L.; Antosiewicz T. J.; Moth-Poulsen K.; Käll M.; Shegai T. Evaluating conditions for strong coupling between nanoparticle plasmons and organic dyes using scattering and absorption spectroscopy. J. Phys. Chem. C. 2016, 120, 20588–20596. 10.1021/acs.jpcc.6b00219.

[ref21] Geng Z.; Theenhaus J.; Patra B. K.; Zheng J.-Y.; Busink J.; Garnett E. C.; Rodriguez S. R. K. Fano lineshapes and Rabi splittings: Can they be artificially generated or obscured by the numerical aperture?. ACS Photonics 2021, 8, 1271–1276. 10.1021/acsphotonics.1c00128. 34056036 PMC8155561

[ref22] Melnikau D.; Esteban R.; Savateeva D.; Sánchez-Iglesias A.; Grzelczak M.; Schmidt M. K.; Liz-Marzán L. M.; Aizpurua J.; Rakovich Y. P. Rabi splitting in photoluminescence spectra of hybrid systems of gold nanorods and J-aggregates. J. Phys. Chem. Lett. 2016, 7, 354–362. 10.1021/acs.jpclett.5b02512. 26726134

[ref23] Renken S.; Pandya R.; Georgiou K.; Jayaprakash R.; Gai L.; Shen Z.; Lidzey D. G.; Rao A.; Musser A. J. Untargeted effects in organic exciton-polariton transient spectroscopy: A cautionary tale. J. Chem. Phys. 2021, 155, 154701 10.1063/5.0063173. 34686047

[ref24] Georgiou K.; Jayaprakash R.; Lidzey D. G. Strong coupling of organic dyes located at the surface of a dielectric slab microcavity. J. Phys. Chem. Lett. 2020, 11, 9893–9900. 10.1021/acs.jpclett.0c02751. 33170714

[ref25] Thomas P. A.; Menghrajani K. S.; Barnes W. L. Cavity-free ultrastrong light-matter coupling. J. Phys. Chem. Lett. 2021, 12, 6914–6918. 10.1021/acs.jpclett.1c01695. 34280306 PMC8327311

[ref26] Runnerstrom E. L.; Kelley K. P.; Sachet E.; Shelton C. T.; Maria J.-P. Epsilon-near-zero modes and surface plasmon resonance in fluorine-doped cadmium oxide thin films. ACS Photonics 2017, 4, 1885–1892. 10.1021/acsphotonics.7b00429.

[ref27] Wang K.; Liu A.-Y.; Hsiao H.-H.; Genet C.; Ebbesen T. Large optical nonlinearity of dielectric nanocavity-assisted Mie resonances strongly coupled to an epsilon-near-zero mode. Nano Lett. 2022, 22, 702–709. 10.1021/acs.nanolett.1c03876. 34994573

[ref28] Passler N. C.; Gubbin C. R.; Folland T. G.; Razdolski I.; Katzer D. S.; Storm D. F.; Wolf M.; De Liberato S.; Caldwell J. D.; Paarmann A. Strong coupling of epsilon-near-zero phonon polaritons in polar dielectric heterostructures. Nano Lett. 2018, 18, 4285–4292. 10.1021/acs.nanolett.8b01273. 29894195

[ref29] Vassant S.; Hugonin J.-P.; Marquier F.; Greffet J.-J. Berreman mode and epsilon near zero mode. Opt. Express 2012, 20, 23971–23977. 10.1364/oe.20.023971. 23188363

[ref30] Berreman D. W. Infrared absorption at longitudinal optic frequency in cubic crystal films. Phys. Rev. 1963, 130, 2193–2198. 10.1103/physrev.130.2193.

[ref31] Newman W. D.; Cortes C. L.; Atkinson J.; Pramanik S.; DeCorby R. G.; Jacob Z. Ferrell-Berreman Modes in Plasmonic Epsilon-near-Zero Media. ACS Photonics 2015, 2, 2–7. 10.1021/ph5003297.

[ref32] Tan W. J.; Thomas P. A.; Luxmoore I. J.; Barnes W. L. Single vs double anti-crossing in the strong coupling between surface plasmons and molecular excitons. J. Chem. Phys. 2021, 154, 024704 10.1063/5.0037864. 33445885

[ref33] Menghrajani K. S.; Fernandez H. A.; Nash G. R.; Barnes W. L. Hybridization of multiple vibrational modes via strong coupling using confined light fields. Adv. Opt. Mater. 2019, 7, 1900403 10.1002/adom.201900403.

