# Supplementary material for: Origin of an Anticrossing between a Leaky Photonic Mode and an Epsilon-Near-Zero Point of Silver
Source: J Phys Chem C Nanomater Interfaces. 2022 Nov 3;126(45):19262–7. doi: 10.1021/acs.jpcc.2c05836 (PMC9677423; doi:10.1021/acs.jpcc.2c05836)
Supplement: Supplementary file 1 — jp2c05836_si_001.pdf [file jp2c05836_si_001.pdf]

Supplementary Information for:

Origin of an Anti-Crossing Between a Leaky Photonic Mode and Epsilon-Near-Zero Point of Silver

Wai Jue Tan*, Philip A. Thomas and William L. Barnes

[1]Department of Physics and Astronomy, University of Exeter,
Exeter, EX4 4QL, United Kingdom

Email: wjt206@exeter.ac.uk

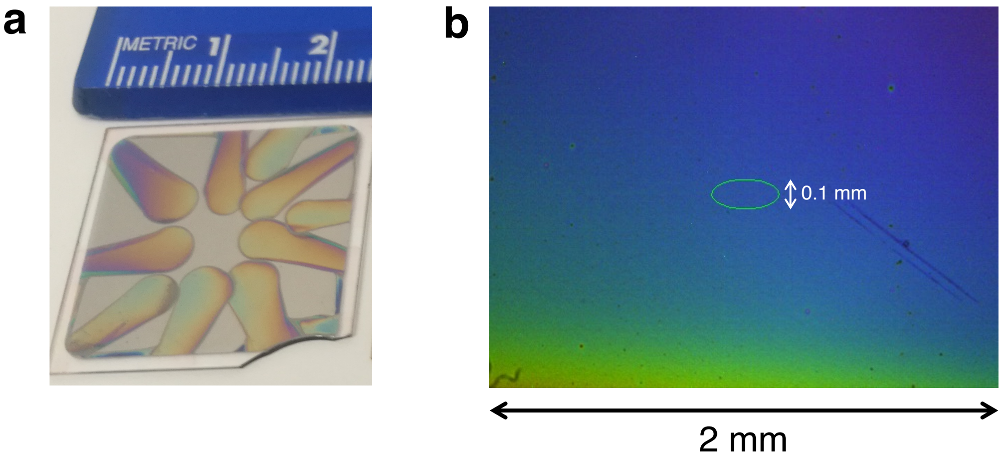

Figure S1: Sample design. (a) Picture of a final dielectric/metal film sample. PMMA was spin-coated off-centre to create a large range of PMMA film thicknesses on a single substrate. (b) Representative close-up image of the surface of the sample. The green ellipse indicates the sample area interrogated in a single measurement at $\theta = 65°$. PMMA films had effectively uniform thickness in a single measured region, allowing us to measure the dispersions of leaky modes (with an excellent match to calculations) in Main Figure 2.

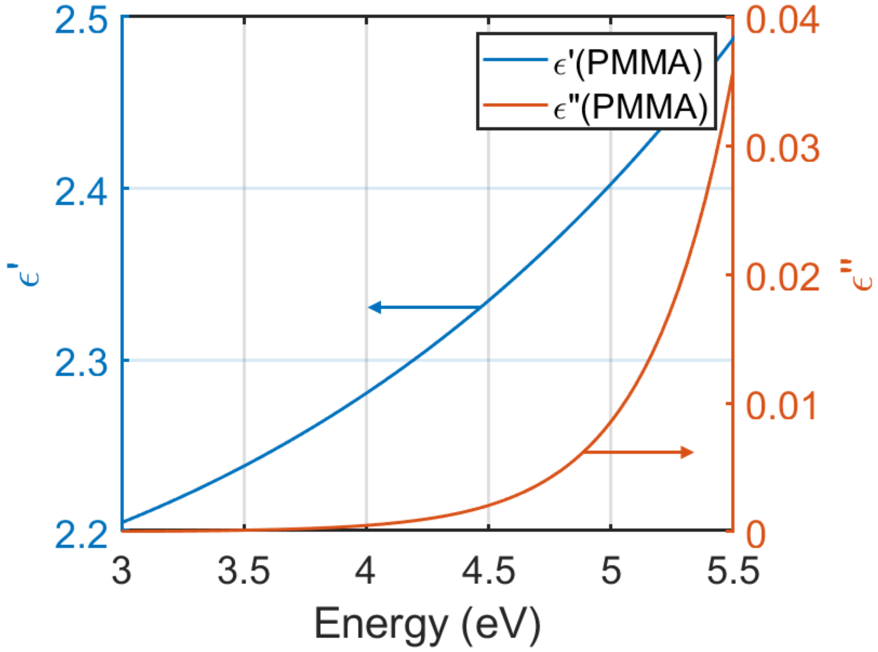

Figure S2: Optical constants of PMMA determined using spectroscopic ellipsometry. A Cauchy dielectic model was used to fit to the PMMA data.

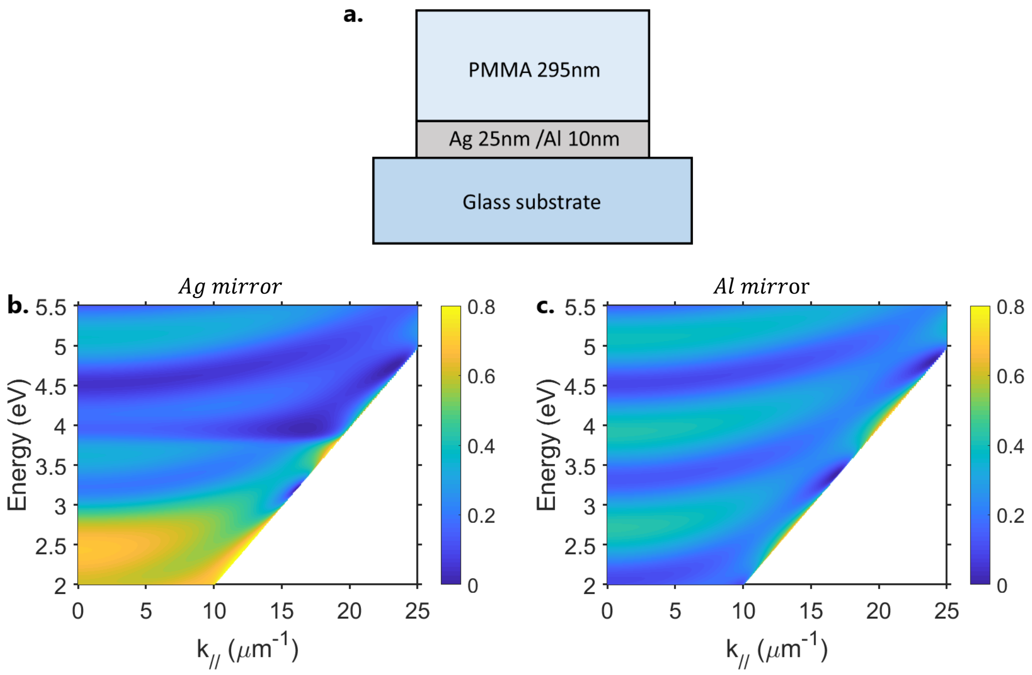

Figure S3: **a.** Schematic of the system studied: Glass/Metal/PMMA(270nm). Calculated TM reflection spectrum of **b.** Glass/Ag(25 nm)/PMMA(295 nm) and **c.** Glass/Al(10 nm)/PMMA(295 nm) as a function of energy and in-plane wave-vector $k_{\|} = \frac{2\pi}{\lambda}\sin\theta$, where λ is the wavelength of light.

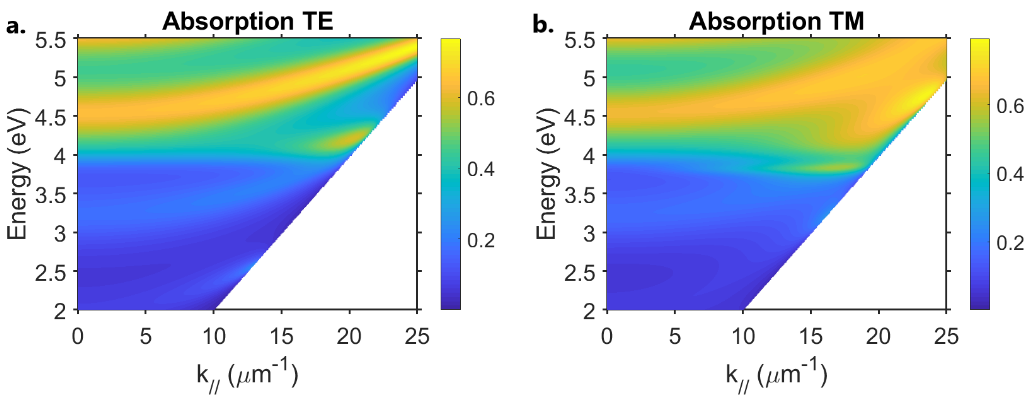

Figure S4: Calculated absorption spectrum of Glass/Ag(25 nm)/PMMA(295 nm) for **a.** TE polarisation and **b.** TM polarisation as a function of energy and in-plane wave-vector $k_{\|} = \frac{2\pi}{\lambda}\sin\theta$, where λ is the wavelength of light.

Similar to the TE reflection spectrum, Glass/Ag(25 nm)/PMMA(295 nm) structure shows a TM reflection minimum at around 4 eV (figure S3**a**). There are two main contributions to this reflection minimum. Same as the TE reflection spectra, the impedance matching between the Ag and PMMA layer at around 4 eV causes the reflection at the Ag/PMMA interface to be minimum. In addition to that, Ag has an ENZ point around 4 eV. Around this energy, the thin Ag film can support a Berreman mode, which results in an enhancement in absorption around the ENZ point, giving it a reflection minimum. This enhancement in absorption can be seen in figure S4**b**.

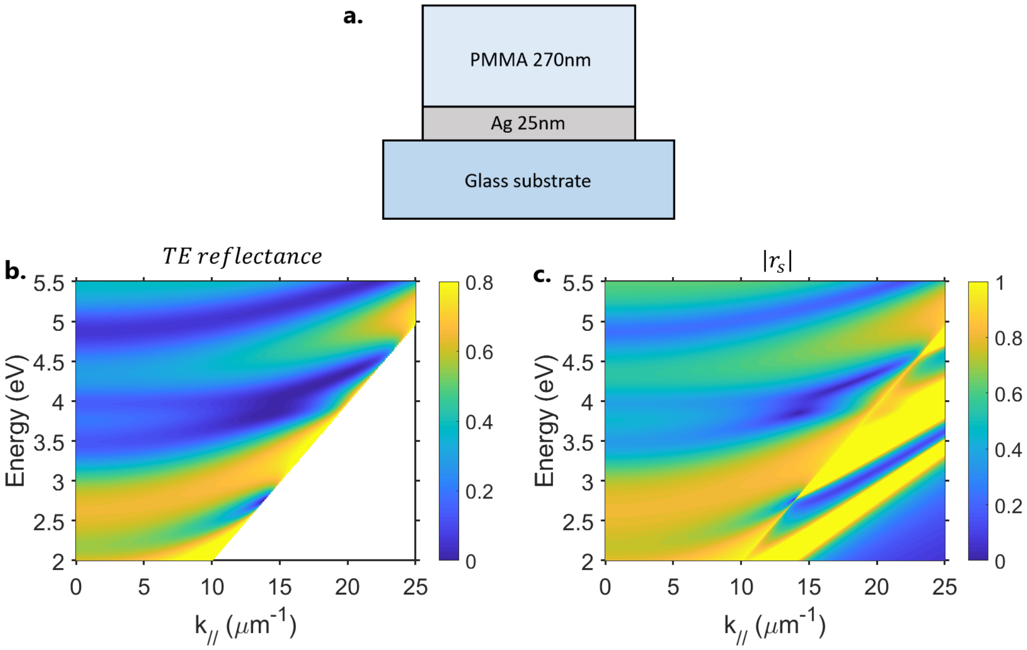

Figure S5: **a.** Schematic of the system studied: Glass/Ag(25nm)/PMMA(270nm). Calculated **b.** TE reflection spectrum and **c.** TE Fresnel reflection amplitude $|r_s|$, of Glass/Ag(25nm)/PMMA(270nm) structure as a function of energy and in-plane wave-vector $k_\parallel = \frac{2\pi}{\lambda}\sin\theta$, where λ is the wavelength of light. $|r_s|$ is plotted here as it has better contrast in showing the anti-crossing.

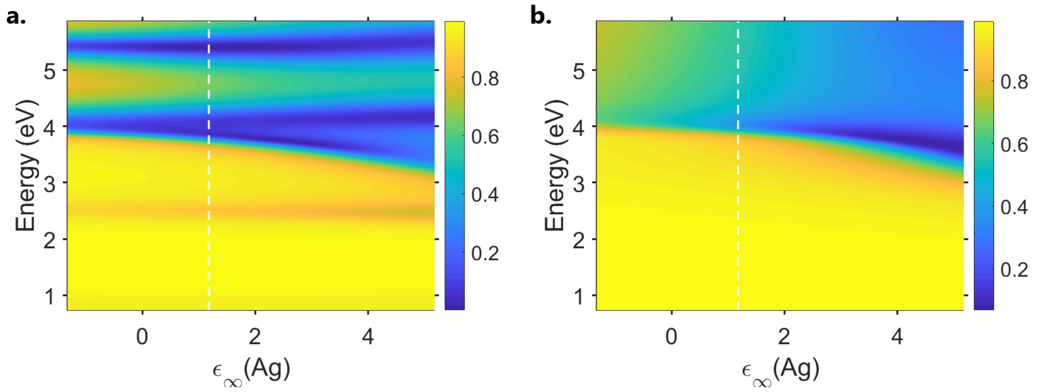

Figure S6: The effect of modifying the background permittivity ϵ_∞ of Ag on the TE reflectivity, calculated for **a.** Ag(semi-infinite)/PMMA(291 nm) structure at $\theta = 65°$ and **b.** Ag/PMMA interface at $\theta = 65°$. The vertical dashed line shows the experimentally-derived value of ϵ_∞ for Ag.

The TE reflectivity spectra of the structure Ag(semi-infinite)/PMMA(291nm) (figure S6**a.** and Ag/PMMA interface (figure S6**b.**) are calculated while varying the background permittivity ϵ_∞, of Ag. The vertical white dashed lines indicate the experimental value of background permittivity $\epsilon_\infty = 1.18$. As the ϵ_∞ increases, the reflectivity minimum of the PMMA/Ag interface around 4eV decreases and moves towards the lower energy. This reflectivity minimum is a result of the impedance matching between the Ag and PMMA layers. It can be seen in figure S6**a.** that the energy where the splitting occurs traces along the reflectivity minimum of figure S6**b.**, showing that the splitting occurs around the energy where the impedance mismatch is minimum.

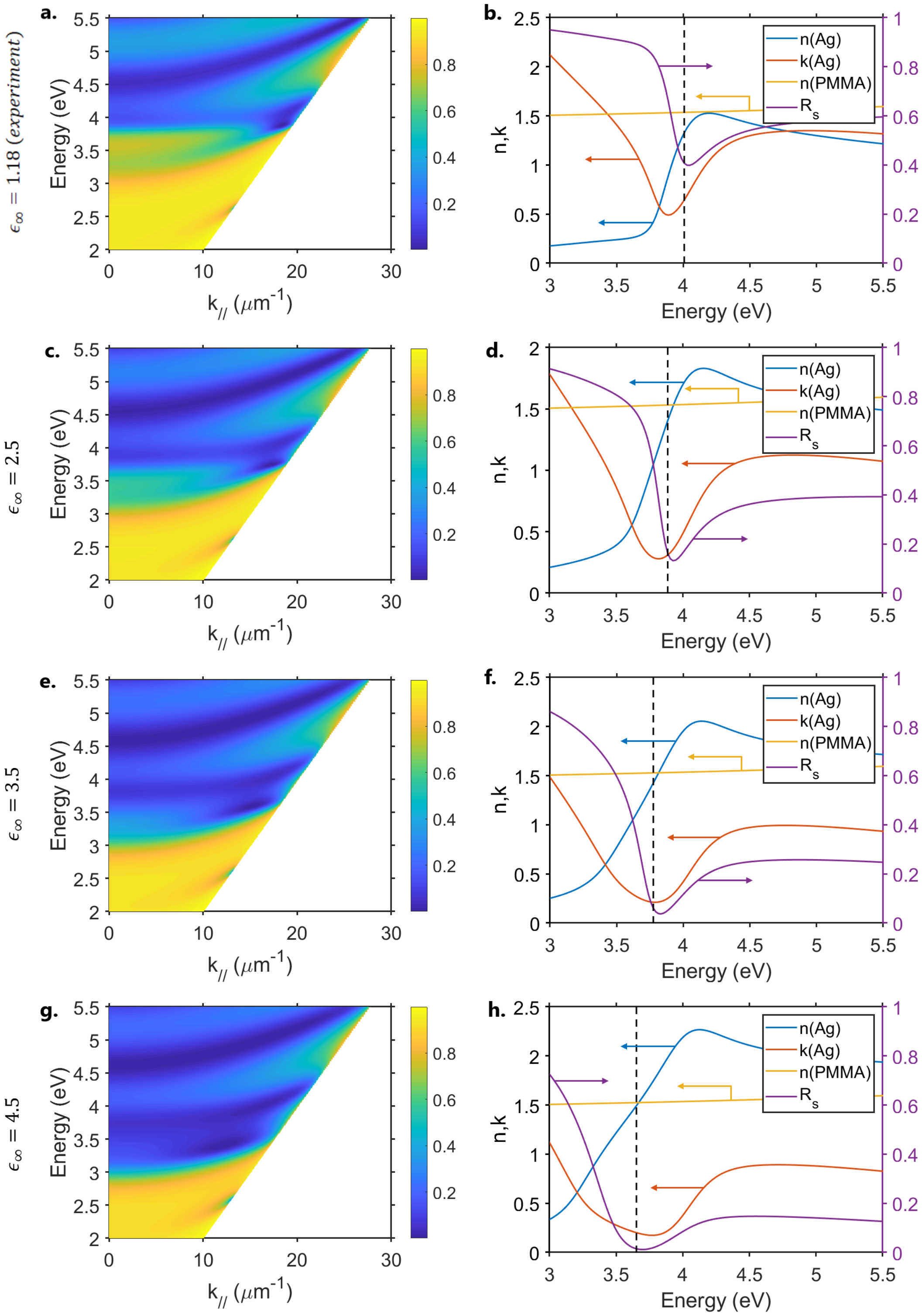

Figure S7: Left-hand column shows the calculated TE reflection spectra of Ag(semi-infinite)/PMMA(291 nm) structure for a different value of ϵ_∞ of Ag. The right-hand column shows the refractive indices of PMMA and Ag, and the reflectance of PMMA/Ag interface at the incident angle where the splitting is minimal. The vertical dashed lines indicate the energies where the splitting occurs.

From figure S7 left-hand column, it can be seen that the energy where the splitting occurs decreases as the ϵ_∞ increases. The size of splitting also appeared to be larger for higher values of ϵ_∞. This is due to the greater impedance matching, as shown in the reflectivity (figure S7, right-hand column), where the reflectivity minimum of PMMA/Ag interface has a lower value when ϵ_∞ is higher. Although refractive indices are not the most accurate parameters to visualize impedance matching

due to their complex nature, they are a good representation of it. It can be seen that around the energy where the splitting happens, the refractive index of Ag closely matches that of the PMMA. The refractive indices do not very well align for the experimental ϵ_∞ case due to the slightly higher value of the imaginary component.
